# High Incidence of a Novel Rickettsia Genotype in Parasitic *Haemaphysalis longicornis* from China-North Korea Border

**DOI:** 10.1038/s41598-019-41879-7

**Published:** 2019-03-29

**Authors:** Heling Xu, Qi Zhang, Hao Guan, Yuening Zhong, Fenghua Jiang, Zeliang Chen, Xiaohu Han

**Affiliations:** 1 Key Laboratory of Zoonotic of Liaoning Province, College of Animal Science and Veterinary Medicine, Shenyang Agricultural University, Liaoning Province, Shenyang 110866 P. R. China; 2 Dandong Animal Disease Control and Prevention Center, Dandong, Liaoning P. R. China

## Abstract

Ticks are notorious vectors for various pathogens that cause infections in animals and humans worldwide. *Rickettsia* spp., a zoonotic tick-borne pathogen that could be used as a weapon agent, is widely spread in China. In the present study, ticks were collected for species identification and *Rickettsia* screening. PCR amplification targeting the tick 18s rRNA gene was first conducted for species validation, and then, amplification was conducted for the *Rickettsia* housekeeping gene for the infection rate and phylogenetic analysis. The collected ticks were identified as *Haemaphysalis longicornis*, 7.36% of which were *Rickettsia*-positive. The phylogenetic analysis showed that the *Rickettsia* in the parasitic ticks belonged to a novel genotype, whose closest genetic relationship was with *Rickettsia heilongjiangenesis*. The samples were collected in Dandong, a city on the border between China and North Korea. Considering the geographical and biological situations of the sampling sites, more extensive surveillance and risk evaluation of the tick species and tick-borne diseases are required.

## Introduction

As hematophagous arthropods, ticks are notorious vectors for various pathogens. Approximately 900 species of ticks have been detected around the world, 36 of which were found in China^1–3^. Ticks can act as vectors, reservoirs, or amplifiers for various pathogens, including viruses, bacteria, *Rickettsia* species, spirochetes, protozoa, mycoplasma, chlamydia, *Bartonella*, and nematodes, to cause infections in animals and humans worldwide^4^. Among these, tick-borne rickettsia, the Xinjiang haemorrhagic fever, Lyme disease caused by spirochetes, and Q fever have been detected in China^5^. *Rickettsia* spp., a pathogen of the spotted fever group, represents an important biological weapon agent, which could cause a great public health crisis^4^. The life cycles of most tick-borne rickettsia are largely unknown. In natural vertebrate hosts, which are mammals, but not normally humans, infections may result in rickettsemia, which allows non-infected ticks to become infected and permits the perpetuation of the natural cycle^6–8^.

Northeast China possesses mountainous terrain and contains abundant biological resources, providing an ecological and biological basis for ticks to survive and reproduce. However, the distribution and infection incidence of these tick species and tick-borne diseases in this area remain unclear. Kuandian Manchu Autonomous County, a large county that is affiliated with Dandong City, southeast of the Liaoning Province, is close to the Democratic People’s Republic of Korea, where the Yalu River acts as the border line. In the present study, ticks were collected from Kuandian County to examine the species and *Rickettsia* harboured.

## Results

### Morphological characteristics and molecular identification of parasitic ticks

**Figure 1:**
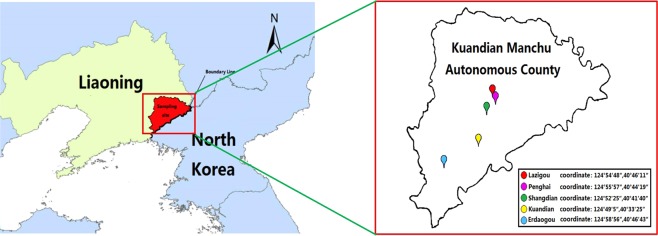
Tick sampling sites in Kuandian County, China-North Korea. The red region represents Kuandian County. The GPS locations of the sampling sites are labelled with their coordinates.

**Figure 2:**
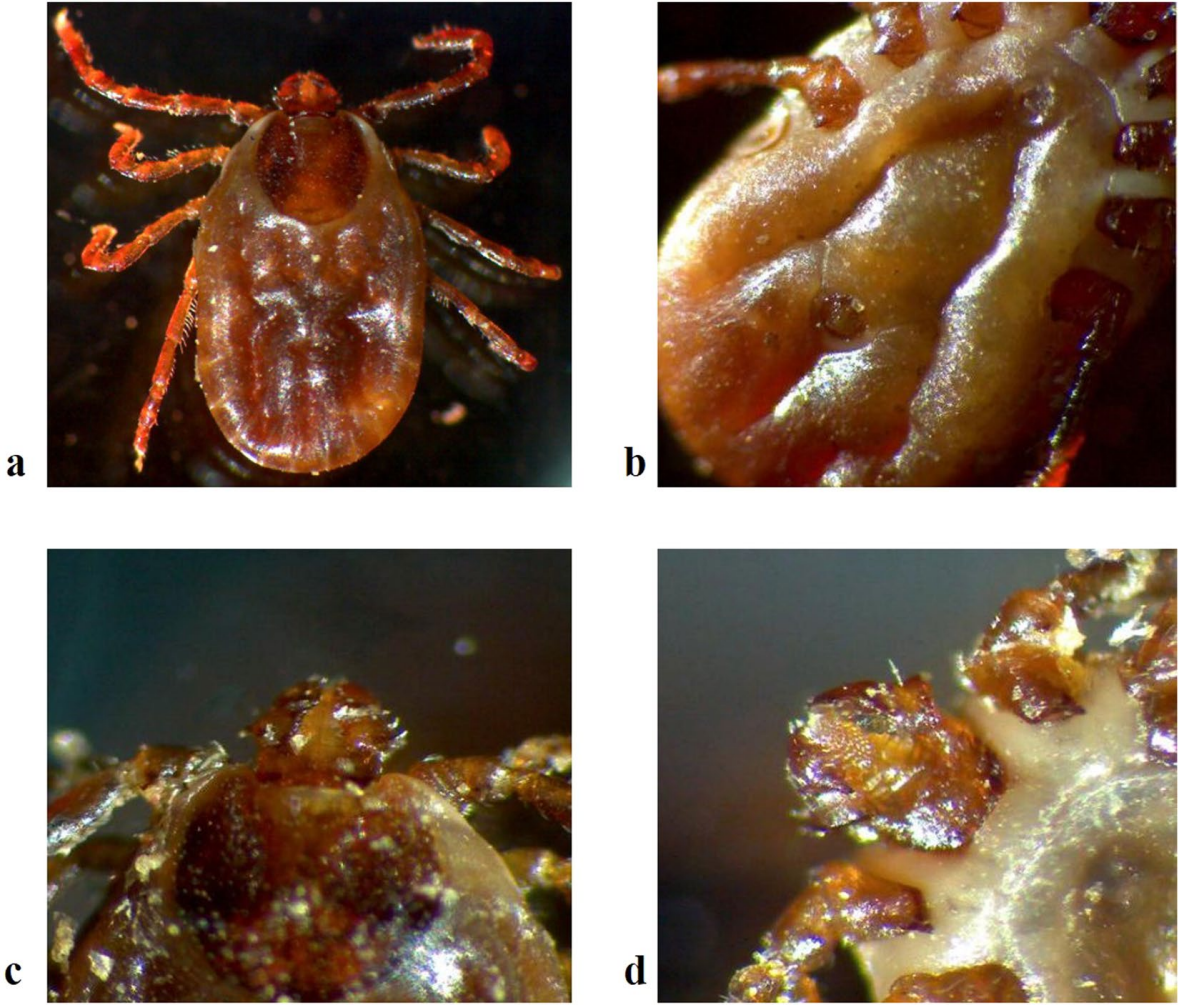
Ticks were observed with a stereomicroscope, and the morphological features of the scutum, venter, and capitulum were recorded. (**a**) The scutum was subcircular, covering the body anteroposteriorly, with clear and slender festoons. Medium-sized punctuations were uniformly and densely distributed. Cervical grooves were short and curved, while the lateral grooves were clear and narrow. The legs were of moderate length with numerous setae. The coxa II had a short but clear ventral spur. (**b**) On the venter, the anal opening was about medioventral with the anal groove. The genital opening was level with the coxa II. The anterior part was a depressed lunula. The spiracular plate was broadly oval and subcircular. On the legs, the coxa I was posterior with a strong cone-shaped spur. (**c**,**d**) The capitulum was short and wedge-shaped. The basis capituli was rectangular was a strong posterior and a triangular cornu. The palpal exterior margin was slightly convex, with corniform present. Five out of five met the definition of hypostome.

Ticks were collected in the Kuandian Manchu Autonomous County, which is located in northeast China and is close to North Korea, along the Yalu River (Fig. 1). About 6000 ticks were collected in 2017 from June 5^th^ to 12^th^. All of these ticks were morphologically identified as *Haemaphysalis longicorni*s (Fig. 2), the representative, marked features of which were as follows. (1) The body was broadly oval or subcircular and cinnamomeous. (2) The capitulum was short and wedge-shaped. The basis capituli was rectangular, with a strong posterior and triangular cornu. The palpal exterior margin was slightly convex, with a coniform present. 5/5 dentition of hypostome. (3) The scutum was subcircular and covered the body anteroposteriorly, with clear and slender festoons. Medium sized punctuations were uniformly and densely distributed. The cervical grooves were short and curved, while the lateral grooves were clear and narrow. (4) On the venter, the anal opening was medioventral with the anal groove. The genital opening was level with the coxa II. The anterior part between the groove was a depressed lunula, while the posterior part was a convex lunula. The spiracular plate was broadly oval or subcircular. (5) The legs were of moderate length with numerous setae. The coxa I was located posteriorly with a strong cone-shaped spur, and the coxa II had a short but clear ventral spur.

**Figure 3:**
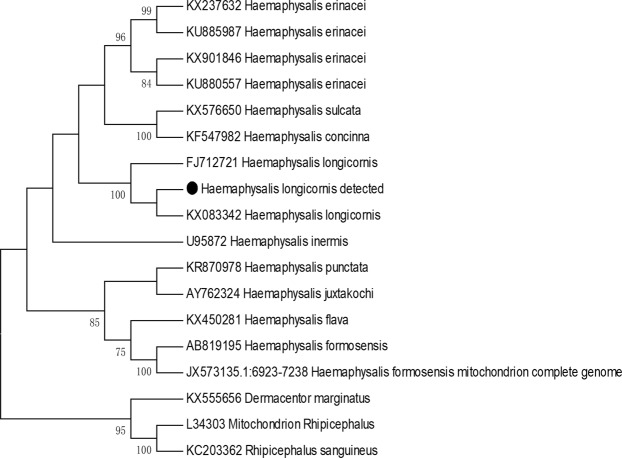
Phylogenetic tree for the representative tick 16 s rDNA gene. The tree was constructed using the maximum likelihood algorithm implemented in the Molecular Evolutionary Genetics Analysis (MEGA) 7 software. The black dot represents the sequence acquired from this study.

The tick DNA was extracted and subjected to PCR amplification. Forty-two of the 489 positive samples (8.5%, selected according to the gender, host, and stage of the tick life cycle) were sent for sequencing of the *rrs* gene, and 40 of which were successfully sequenced. The sequences from all of the samples were completely identical. The phylogenetic tree for the *rrs* gene showed that these ticks were closest to *Haemaphysalis longicornis* (Fig. 3). Combined with the morphological characteristics and molecular evidence, these ticks were classified as *H*. *longicornis*.

### Molecular identification and genotypes of *Rickettsia*

**Figure 4:**
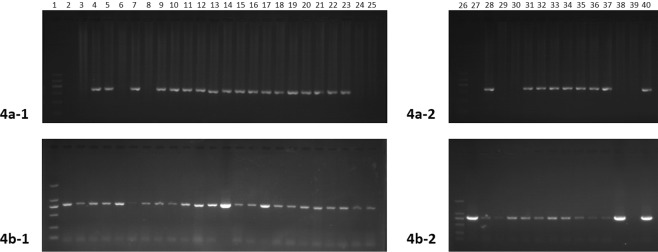
PCR amplification results for the *Rickettsia*-positive samples. Gene amplification of *ompA* (**a**) and *ompB* (**b**). For both figures, Lanes 1 and 26: Markers; Lanes 2–14: *gltA*-positive samples from the village of Erdaogou; Lanes 15–22: *gltA*-positive samples from the village of Kuandian; Lanes 23–25 and 27–29: *gltA*-positive samples from the village of Lazigou; Lanes 30–35: *gltA*-positive samples from the village of Shangdian; Lanes 36–38: *gltA*-positive samples from the village of Penghai; Lane 39: negative control; and Lane 40: positive control. All positive samples were sent for sequencing.

**Figure 5:**
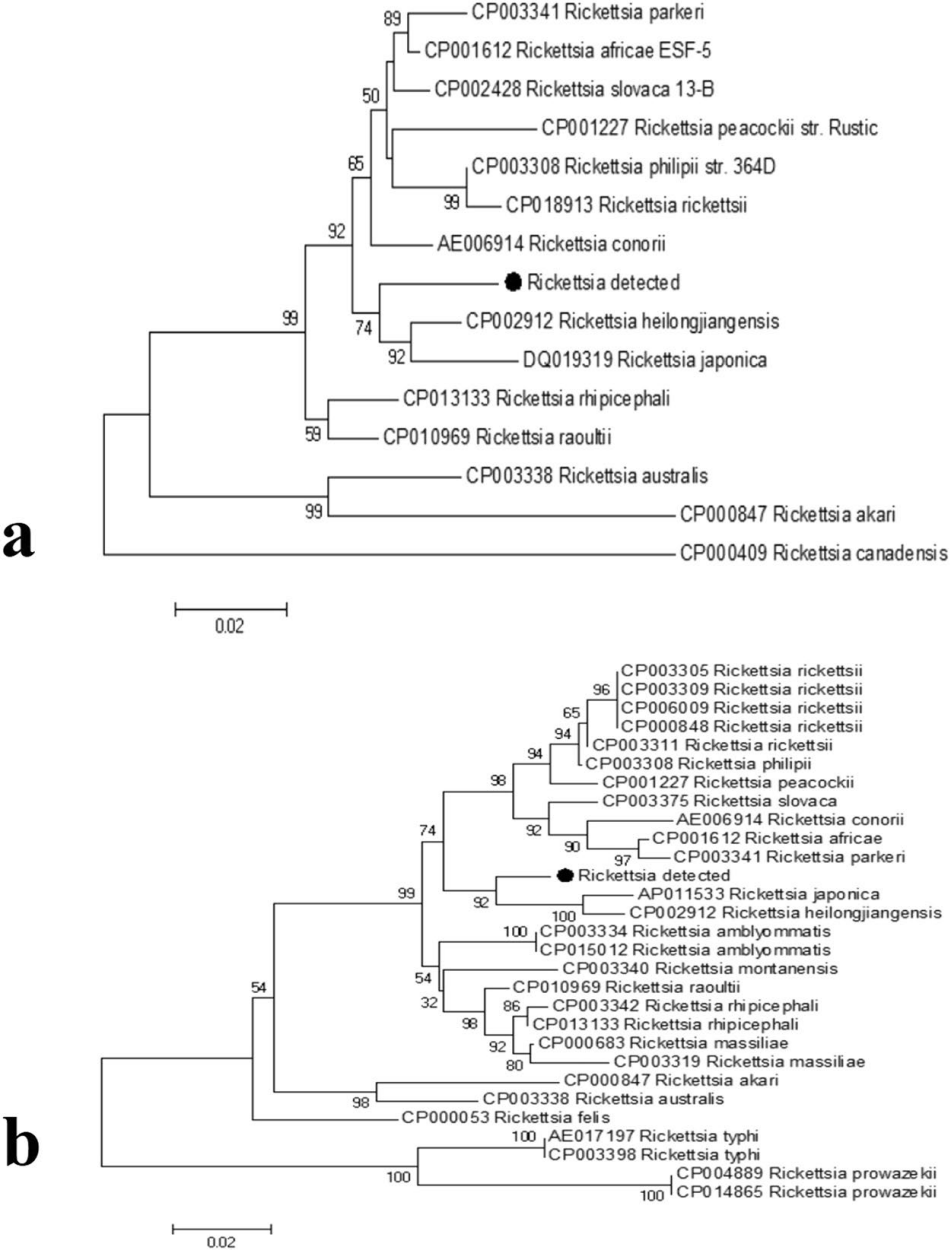
Phylogenetic trees for the *ompA* and *ompB* genes. The trees were constructed using the sequences of *ompA* (**a**) and *ompB* (**b**), using the maximum likelihood (ML) algorithm implemented in the Molecular Evolutionary Genetics Analysis (MEGA) 7 software. The black dots represent the sequences acquired from this study.

**Figure 6:**
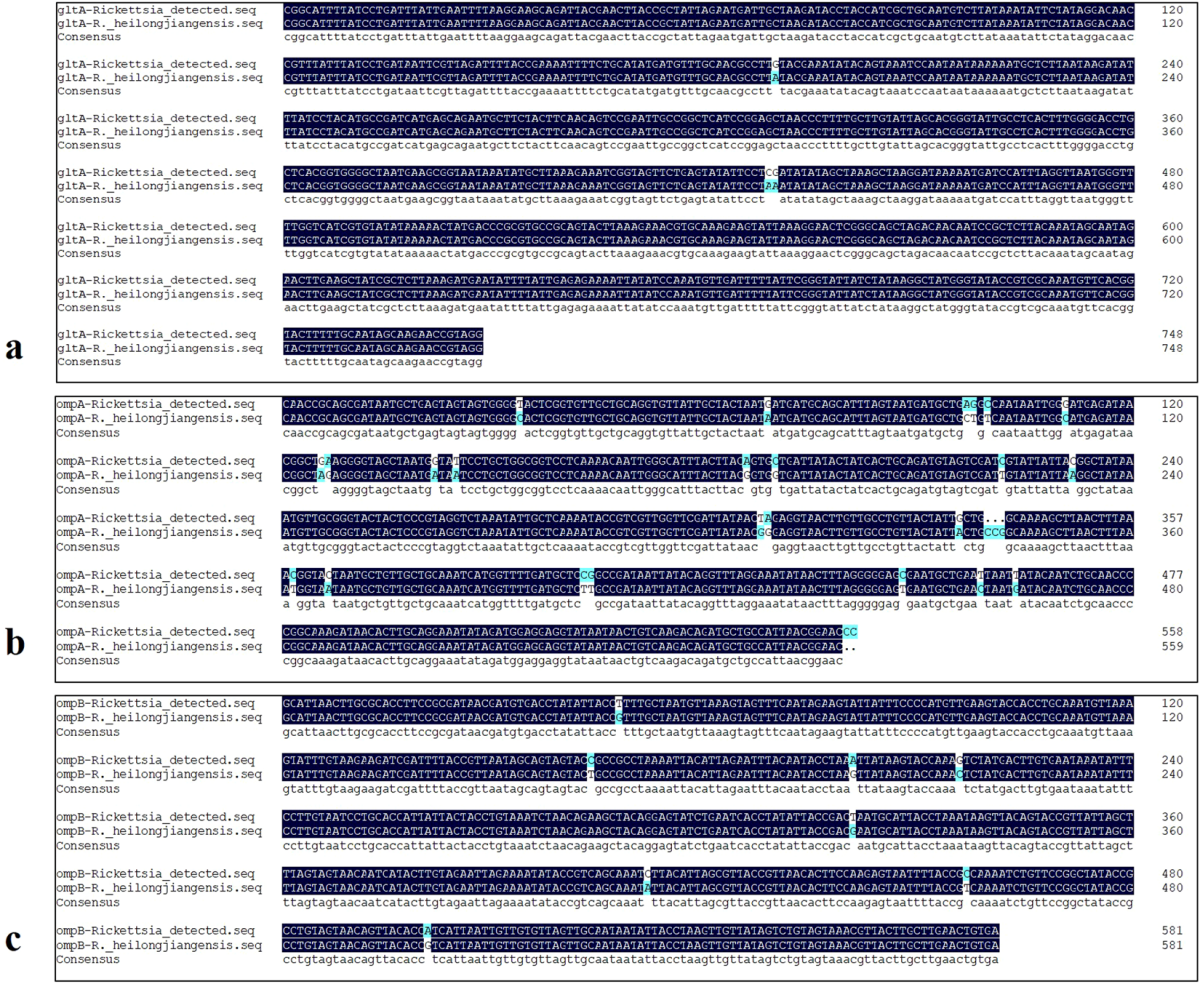
Sequence alignment between the *Rickettsia* detected in this study and *R*. *heilongjiangensis*. The sequence alignments for *gltA*, *ompA*, and *ompB* were conducted using the Multiple Sequence Alignment in the DNAMAN software.

Genomic DNA was extracted from representative tick samples and subjected to PCR amplification of the *gltA* gene. Among the 489 tested samples, 36 were *gltA* positive, generating an average positive rate of 7.36%. The *Rickettsia*-positive rate for each sampling site was calculated. The positive rates for these sites ranged from 4.10% to 9.22%, with the village of Erdaogou (9.22%) having the highest, followed by Kuandian (7.92%) and Shandian (7.79%); whereas, the positive rates for Lazigou (6.19%) and Penghai (4.10%) were relatively low for all of the collection sites (Table 1). The 36 *Rickettsia*-positive DNA samples were then amplified and sequenced for both *ompA* and *ompB*, which are two commonly used genotyping genes. The PCR amplification results showed that 27 of the 36 samples were *ompA* positive, and 32 were *ompB* positive; 24 (66.7%) were positive for both genes (Fig. 4). All of the PCR products from the positive samples (35 in total) were sent for sequencing. The sequences for each gene from all of the samples were completely identical. The phylogenetic tree showed that the *Rickettsia* detected herein belongs to a novel genotype (Fig. 5). According to the criteria for novel *Rickettsia* determination based on gene sequences, an isolate can be classified as a new *Rickettsia* genotype when it exhibits no more than one of the following degrees of nucleotide similarity with the most homologous validated species: ≥99.8% and ≥99.9% for the *rrs* and *gltA* genes, respectively, and, when amplifiable, ≥98.8%, ≥99.2%, and ≥99.3% for *ompA*, *ompB*, and gene *D*, respectively^9^. Thus, we made a sequence alignment based on our sequenced genes, *gltA*, *ompA* and *ompB*, between the *Rickettsia* detected in this study and *R*. *heilongjiangensis* or *R*. *japonica*. The results of the alignment with *R*. *heilongjiangensis*, the most homologous validated species, are shown in Fig. 6, for which the percentages for the nucleotide identities were 99.6% for the *gltA* gene (Fig. 6a), 94.66% for the *ompA* gene (Fig. 6b), and 98.63% for the *ompB* gene (Fig. 6c). The sequence alignment with R. japonica was showed in Figure S1. Even though the sequence alignment results were promising, the evidence to prove that the newly detected *Rickettsia* is a novel *Rickettsia* species was insufficient. Therefore, we temporarily determined that the detected rickettsiae is a novel genotype.

**Table 1:** The positive rate for each collection site.

Location	Tick species	Tested number	Positive number	Positive rate
Erdaogou	Haemaphysalis longicornis	141	13	9.22%
Kuandian	Haemaphysalis longicornis	101	8	7.92%
Lazigou	Haemaphysalis longicornis	97	6	6.19%
Shangdian	Haemaphysalis longicornis	77	6	7.79%
Penghai	Haemaphysalis longicornis	73	3	4.10%

## Discussion

In the present study, ticks, collected from the Liaoning province, were morphologically and phylogenetically shown to be *Haemaphysalis longicornis*, which has been detected in both animals and humans and as a host for various kinds of emerging pathogens. For instance, in China, during the past 15 years, *H*. *longicornis* has been found in: (1) the Heilongjiang and Jilin provinces, along with *Haemaphysalis concinna*, *Dermacentor nuttalli*, *Dermacentor silvarum*, and *Ixodes persulcatus*, conveying *Rickettsia* spp.^10^; (2) in the Hebei province, conveying the spotted fever group Rickettsia^11^; (3) in the Henan province, along with *Rhipicephalus microplus*, conveying *Anaplasma* spp., *Rickettsia* spp., *Babesia* spp., *Theileria* spp., *Ehrlichia* spp., and severe fever with thrombocytopenia syndrome bunyavirus (SFTSV)^12,13^; and (4) in the Zhejiang province, along with *Amblyomma testudinarium* and *Ixodes sinensis*, conveying *Rickettsia* spp.^14^. Other studies from South Korea, Japan, New Calenonia, and New Zealand have also illustrated the extensive distribution and high incidence of ticks and tick-borne diseases^15–21^. Close attention has been paid to the geographic distribution of ticks, as tick-borne diseases put the public health at risk. According to the review by Chen^1^, at least 104 species from 7 genera of ticks have been circulated in China, but the ticks collected in the present study were homogenous. Therefore, the distribution of ticks in the Liaoning province remains unclear and needs further investigation.

The *Rickettsia*-positive rate among all the specimens was 7.36% (36/489). The *Rickettsia* we detected have the closest genetic relationship with *R*. *heilongjiangenesis*, but they are located on a separate branch, implying that they might belong to a novel genotype. The qualification of new a *Rickettsia* genotype remains to be further defined. As a zoonotic disease, at least five species of spotted fever group *Rickettsia* have been detected in China, including *R*. *heilongjiangenesis*, *Rickettsia sibirica*, *Rickettsia raoultii*, *Rickettsia slovaca*, and *Rickettsia felis*^4,22^. In previous studies, the *Rickettsia*-positive rate in Xinyang in the Henan province (6.25%, 9/144) and in the Jilin and Heilongjiang provinces (6.12%) was about 6%^10,12,23^. Compared to these regions in China, the *Rickettsia*-positive rate at our sampling site, the national border line in the Liaoning province, was relatively high. This high positive rate implies a high infection risk and a potential public health problem. *Rickettsia* is mutually transmitted through arthropods and humans, representing an important pathogen that is circulated by ticks. With their high prevalence and species diversity, as well as the geographical spectrum, ticks in northeast China may be able to transmit numerous pathogens, causing potential severe infections in both animals and humans. In the sampling regions, livestock are mainly bred by free range. This sharply increases the possibility for human-tick-livestock contact and transmission. However, neither tick species, nor tick-borne diseases, have been thoroughly investigated in these regions.

Although our current investigation on ticks and tick-borne diseases at the China-North Korea border is not completely comprehensive, the results we obtained do have several implications. First, unlike the research in other provinces in north-eastern China (the Heilongjiang and Jilin provinces)^10^, all 489 ticks collected in the Liaoning province were *H*. *longicornis*, indicating that *H*. *longicornis* is the predominant tick species in the sampling region. Furthermore, the identified *Rickettsia* genotype is also different from those observed during previous research studies in northeast China. Second, because the sampling site was only located along the border area, the distribution of both the ticks and the *Rickettsia* in other regions of the Liaoning province remains unknown. Likewise, it is of great interest to determine whether *H*. *longicornis* and the novel *Rickettsia* genotype also circulate. Third, further investigation into the distribution of *Rickettsia* and other vector-borne pathogens at the border line is important for risk evaluation for emerging infectious diseases. In our ongoing work, we are trying to widen the sampling spectrum for other tick species and tick-borne pathogens and to investigate the distribution of the novel *Rickettsia* genotype in different arthropods in this region. These studies will greatly contribute to our understanding of ticks and tick-borne diseases and will allow us to evaluate the circulating risk.

## Methods

### Tick sampling

The sampling cites were located in the Kuandian Manchu Autonomous County. The altitudes and latitudes of sampling sites are shown in Table 2. Ticks were manually collected from animal skin without damage and then carefully placed into 70% ethanol. Each sample was labelled with the area name, latitude, longitude, host type, and the collection date and site. All specimens were then sent to the laboratory at the Shenyang Agricultural University and stored for further examination.

**Table 2:** The location coordinates.

Village	Longitude	Latitude
Lazigou	124°54′48″	40°46′11″
Penghai	124°55′57″	40°44′19″
Shangdian	124°52′25″	40°41′40″
Kuandian	124°49′5″	40°33′25″
Erdaogou	124°58′56″	40°46′43″

### Morphological identification

The morphological features of the ticks were individually observed by a stereomicroscope. The back, abdomen, shield plate, gas door plate, false head base, lateral furrow, and genital orifice were carefully examined for species identification.

### Molecular identification

The protection solution was removed, and the specimens were vortexed and rinsed with 75% ethanol for 1 h. Then, they were rinsed with distilled water three times. The DNA was extracted using a DNA extraction kit (Tiangen Biochemical Technology (Beijing) Co., Ltd.), according to the manufacturer’s protocols, and stored at −20 °C. PCR amplification was first conducted with paired universal primers that targeted the 18S rRNA gene (*rrs*) for tick species and then the *Rickettsia* citrate synthase gene (*gltA*) to calculate the positive rate^24^. The *Rickettsia*-positive samples were then passaged for the detection of two more genes: outer membrane protein A (*ompA*) and B (*ompB*)^3,9^. Double distilled water and the recombined plasmids of each gene were used as the negative and positive controls, respectively. The primer sequences are shown in Table 3 ^24^. All PCR products were sequenced by Sanger sequencing (Sangon Biotech Co., Ltd, Shanghai, China). Related *Rickettsia* sequences were extracted from the Genbank database and edited along with the generated sequences. The phylogenetic tree was made based on the sequence distance method using the neighbor-joining (NJ) and maximum likelihood (ML) algorithms implemented in the Molecular Evolutionary Genetics Analysis (MEGA) 7 software^25^.

**Table 3:** Primers used in the study.

Gene	Primer/probe	Sequences(5′-3′)	Reference
16S rRNA	16S-F	CTGCTCAATGATTTTTTAAATTGCTGTGG	^16^
	16S-R	CCGGTCTGAACTCAGATCAAGT	
gltA	Cs-239	GCTCTTCTCATCCTATGGCTATTAT	^17^
	Cs-1069	CAGGGTCTTCGTGCATTTCTT	
ompA	RcromA190-70	ATGGCGAATATTTCTCCAAAA	^18^
	RcromA190-71	GTTCCGTTAATGGCAGCATCT	
ompB	ompB-1	TACTTCCGGTTACAGCAAAGT	^19^
	ompB-2	AAACAATAATCAAGGTACTGT	

All animal sampling operations were performed properly according to the protocols proposed by NEON and AfiVIP: *TOS Protocol and Procedure: Tick and Tick-borne Pathogen Sampling* and *Ticks: Tick surveillance*. The morphological and molecular identification was performed in the Biosafe Laboratories of Shenyang Agricultural University using the relevant equipment with formal approval.

## Supplementary information

Figure S1
