# Supplementary material for: High Incidence of a Novel Rickettsia Genotype in Parasitic Haemaphysalis longicornis from China-North Korea Border
Source: Sci Rep. 2019 Mar 29;9:5373. doi: 10.1038/s41598-019-41879-7 (PMC6440996; doi:10.1038/s41598-019-41879-7)

High Incidence of a Novel Rickettsia Genotype in Parasitic *Haemaphysalis longicornis* from China-North Korea Border

Heling Xu[1, #], Qi Zhang[1, #], Hao Guan[1, #], Yuening Zhong[1], Fenghua Jiang[2], Zeliang Chen[1, *], Xiaohu Han[1, *]

[1] Key Laboratory of Zoonotic of Liaoning Province, College of Animal Science and Veterinary Medicine, Shenyang Agricultural University, Liaoning Province, Shenyang 110866, P. R. China
[2] Dandong Animal Disease Control and Prevention Center, Dandong, Liaoning, P. R. China

# the authors contributed equally to this work
* Correspondence to Z. Chen [zeliangchen@yahoo.com] or X. Han [hxh8849@163.com]

Supplementary Dataset

Figure S1. Sequence alignment between the *Rickettsia* detected in this study and *R. japonica*. The sequence alignments for *gltA*, *ompA*, and *ompB* were conducted using the Multiple Sequence Alignment in the DNAMAN software.

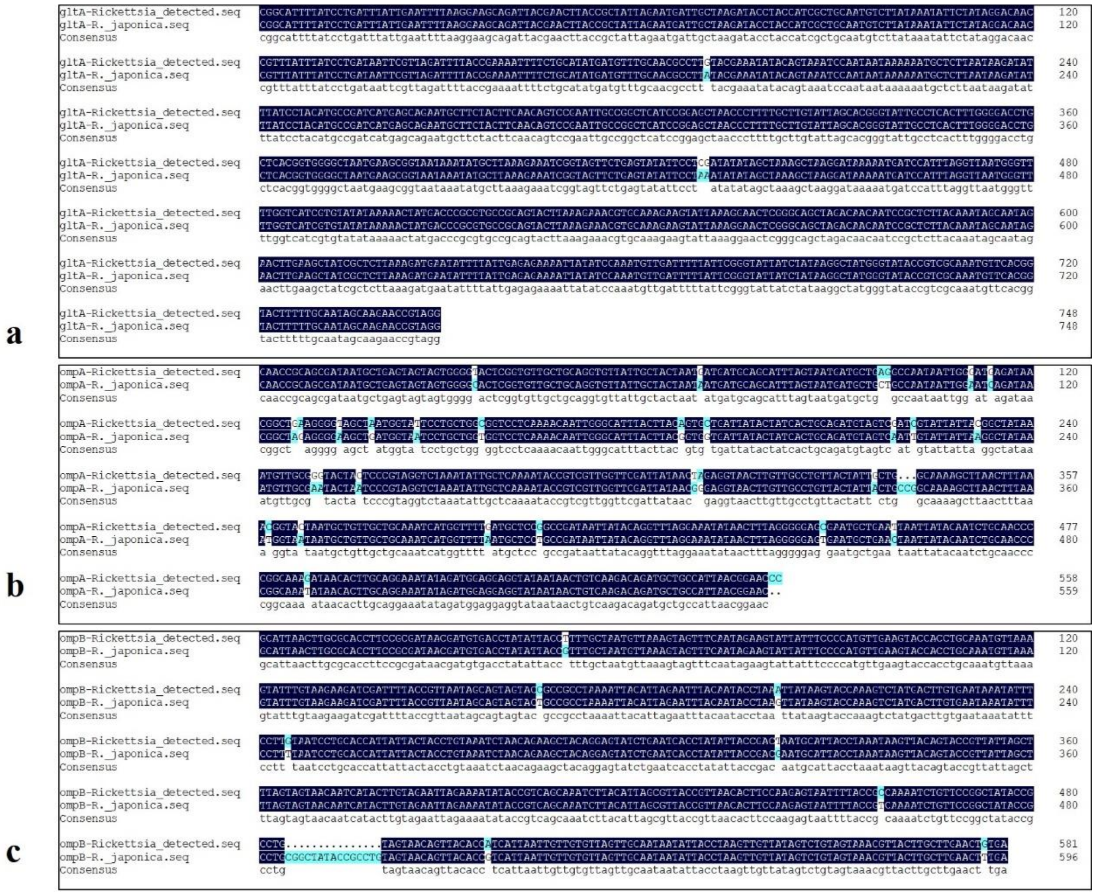

Supplement: Supplementary file 1 — Figure S1 [file 41598_2019_41879_MOESM1_ESM.pdf]
